# CXCL17 Attenuates Diesel Exhaust Emissions Exposure-Induced Lung Damage by Regulating Macrophage Function

**DOI:** 10.3390/toxics11080646

**Published:** 2023-07-26

**Authors:** Yize Yin, Chaohui Mu, Jiahui Wang, Yixuan Wang, Wenmin Hu, Wenjing Zhu, Xinjuan Yu, Wanming Hao, Yuxin Zheng, Qinghai Li, Wei Han

**Affiliations:** 1 Department of Pulmonary and Critical Care Medicine, Qingdao Municipal Hospital, School of Public Health, Qingdao University, Qingdao 266071, China; jingzhechuri@163.com; 2 Department of Pulmonary and Critical Care Medicine, Qingdao Municipal Hospital, School of Medicine, Qingdao University, Qingdao 266071, China; MCH0580@163.com; 3 Department of Respiratory and Critical Care Medicine, Qingdao Municipal Hospital, University of Health and Rehabilitation Science, Qingdao 266071, China; zoewjh@126.com (J.W.); 13583205903@163.com (W.H.); 4 Central Laboratories and Department of Gastroenterology, Qingdao Municipal Hospital, Qingdao 266071, China; yixuanwang@uor.edu.cn; 5 Respiratory Disease Key Laboratory of Qingdao, Qingdao Municipal Hospital, Qingdao University, Qingdao 266071, China; 15628708593@163.cm (W.Z.); yxj4501@126.com (X.Y.); 6 School of Medicine and Pharmacy, Ocean University of China, Department of Pulmonary and Critical Care Medicine, University of Health and Rehabilitation Science, Qingdao 266071, China; huwenmin1997@163.com; 7 Clinical Research Center, Qingdao Municipal Hospital, University of Health and Rehabilitation Science, Qingdao 266071, China; 8 Department of Occupational and Environmental Health, School of Public Health, Qingdao University, Qingdao 266071, China; yx_zheng@139.com

**Keywords:** diesel engine exhaust, macrophages, C-X-C motif chemokine ligand 17, human bronchial airway epithelial cells

## Abstract

Exposure to diesel exhaust emissions (DEE) is strongly linked to innate immune injury and lung injury, but the role of macrophage chemoattractant CXCL17 in the lung damage caused by DEE exposure remains unclear. In this study, whole-body plethysmography (WBP), inflammatory cell differential count, and histopathological analysis were performed to assess respiratory parameters, airway inflammation, and airway injury in C57BL/6 male mice exposed to DEE for 3 months. qRT-PCR, IHC (immunohistochemistry), and ELISA were performed to measure the CXCL17 expression in airway epithelium or BALF (bronchoalveolar lavage fluid) following DEE/Diesel exhaust particle (DEP) exposure. Respiratory parameters, airway inflammation, and airway injury were assessed in CXCL17-overexpressing mice through adeno-associated virus vector Type 5 (AAV5) infection. Additionally, an in vitro THP-1 and HBE co-culture system was constructed. Transwell assay was carried out to evaluate the effect of rh-CXCL17 (recombinant human protein-CXCL17) on THP-1 cell migration. Flow cytometry and qRT-PCR were conducted to assess the impacts of rh-CXCL17 on apoptosis and inflammation/remodeling of HBE cells. We found that the mice exposed to DEE showed abnormal respiratory parameters, accompanied by airway injury and remodeling (ciliary injury in airway epithelium, airway smooth muscle hyperplasia, and increased collagen deposition). Carbon content in airway macrophages (CCAM), but not the number of macrophages in BALF, increased significantly. CXCL17 expression significantly decreased in mice airways and HBE after DEE/DEP exposure. AAV5-CXCL17 enhanced macrophage recruitment and clearance of DEE in the lungs of mice, and it improved respiratory parameters, airway injury, and airway remodeling. In the THP-1/HBE co-culture system, rh-CXCL17 increased THP-1 cell migration while attenuating HBE cell apoptosis and inflammation/remodeling. Therefore, CXCL17 might attenuate DEE-induced lung damage by recruiting and activating pulmonary macrophages, which is expected to be a novel therapeutic target for DEE-associated lung diseases.

## 1. Introduction

With unprecedented urbanization and industrialization worldwide, air pollution as an emerging anthropogenic pollutant has been the most distinctive feature of this era and one of the most important public health problems, resulting in approximately 9-million deaths annually. According to the air quality limits proposed by WHO (PM2.5 < 25 μg/m^3^), approximately 90% of the global population lives in areas that exceed the air quality limits, with more severe problems in developing countries [1]. In China, 43.1% of the 339 major cities surveyed in 2021 did not meet our relatively relaxed air quality limits (China PM2.5 concentration limit: 75 μg/m^3^) according to the 2021 China Ecological and Environmental Quality Bulletin. It has been reported that air pollution is the fourth-leading risk factor for mortality and disability-adjusted life years in China [2]. Diesel exhaust emissions (DEE), a major contributor to air pollution in the environment, are a complex mixture of gases (such as NO, CO, and NO_2_) and particulate matter (such as diesel exhaust particles (DEP)), and their main component is the carbonaceous core with polycyclic aromatic hydrocarbons (PAH) and heavy metals adsorbed to their surface [3,4,5]. As the main target organ of DEE, the respiratory system will show increased inflammatory cell infiltration, epithelial cell transformation and apoptosis, collagen deposition, and goblet cell hyperplasia, which can trigger chronic obstructive pulmonary disease (COPD), asthma, and even lung cancer [6,7]. However, the exact causal relation between DEE and harmful effects on health remains unclear.

After DEE passes through the upper respiratory tract, considerable particulate matter components will be deposited in the lungs, while macrophages act as the first line of innate immune defense that can phagocytose and clear these components to mitigate local inflammation and damage. However, it has been reported that the phagocytic rate and phagocytic index of macrophages are significantly reduced after DEP exposure [8,9,10,11]. For instance, DEP can form aggregates of various sizes and hinder the function of alveolar macrophages, which participates in the development of multiple respiratory diseases [12]. Although studies have indicated that regulation of macrophage polarization can alleviate lipopolysaccharide (LPS)-induced lung injury [13,14], no study has been conducted to ameliorate DEE-related lung injury by augmenting the number and function of macrophages.

As a newly discovered member of the CXC chemokine family, CXCL17 is specifically and highly expressed in the respiratory tract mucosa, and it can recruit or activate monocytes/macrophages and dendritic cells, which can affect the strength and quality of innate immunity [15,16,17,18,19]. Burkhardt has found that alveolar macrophages were significantly reduced in CXCL17^−/−^ mice, whereas no differences were noted in macrophage subsets in other tissues [20]. It is suggested that CXCL17 is closely related to the recruitment of lung macrophages, but its role in DEE-induced lung injury remains undefined. In this study, we intended to determine the CXCL17 expression in airway epithelium after DEE exposure, as well as its role and possible mechanism in DEE-caused lung injuries.

## 2. Materials and Methods

### 2.1. Animals and Ethics Considerations

Animal experiments were conducted using 8-week-old C57BL/6 male mice from Jinan Pengyue Laboratory Animal Breeding Ltd. (Jinan, China). Mice were housed in a room with a relative humidity of 50–60%, a temperature of 24–26 °C, a 12/12 h light/dark cycle, and free access to food and water. Experimental animal procedures were approved by the Animal Ethics Committee of Qingdao Municipal Hospital.

### 2.2. Exposure Chamber and Characterization

The diesel exhaust was produced using a model 192 single-cylinder diesel generator (a gift from Prof. Yuxin Zheng, Qingdao University; a load of 4000 kW) with 0# diesel purchased from Qingdao City and diluted to the required concentration by connecting to a clean air pump. In addition, the diesel generator was placed in a silent chamber. Two devices were installed in an exposure chamber connected to the diesel generator to record pollutant parameters: one (CASELLA, England) for PM2.5 concentration and another (LOOBO, China) for monitoring the NO_2_ and NO concentrations (Supplementary Table S1).

### 2.3. Study Design

**Figure 1:**
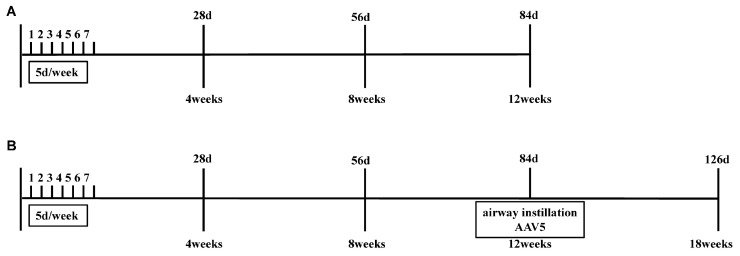
Schematic drawing of the experimental design. (**A**) Model construction. (**B**) Animal intervention.

Model construction: mice were randomly assigned to control group (n = 6) and DEE group (n = 6). The mice in the DEE group were exposed to 3 mg/m^3^ DEE for 1 h per day, 5 days per week for 3 months [6,21,22], while the mice in the control group were exposed to clean air (Figure 1A).

Animal intervention: mice were randomly divided into control group (n = 6), DEE group (n = 6), and DEE + CXCL17 group (n = 6). The mice in the DEE and DEE + CXCL17 groups were exposed to 3 mg/m^3^ DEE for 1 h per day, 5 days per week for 3 months, while the mice in the control group were exposed to clean air. After 90 days, the mice in the DEE group and DEE + CXCL17 group were infected by airway instillation with an empty vector adeno-associated virus Type 5 (AAV5-GFP, 5 × 10^10^ vg/mouse) and adeno-associated virus Type 5 overexpressing CXCL17 (AAV5-CXCL17, 5 × 10^10^ vg/mouse), respectively. Following infection, the mice were exposed to DEE for another 6 weeks (Figure 1B).

### 2.4. Respiratory Parameter Evaluation

Respiratory parameter evaluation of mice was conducted using whole-body plethysmography (Whole-body Plethysmography System, Tow-Int Tech, Shanghai, China). Awake mice capable of spontaneous breathing were placed in the whole-body plethysmography chamber, and their respiratory parameters were measured after falling asleep.

### 2.5. Collection of Blood, Bronchoalveolar Lavage Fluid (BALF), and Lungs

Twenty-four hours after the final expose, the animals were anesthetized with intra-peritoneal injection of pentobarbital (50 mg/kg). Blood was collected from the abdominal aorta and mice were euthanized. Blood was stored at 4 °C for 2 h. The plasma was isolated by centrifugation at 1000 rpm for 10 min and stored at −80 °C. The thoracic cavity was opened, and the main bronchus of the right lung was ligated. The left lung was lavaged for 3 times after intubation and BALF was collected. BALF was centrifuged at 300× *g* for 10 min, and the cell pellet and supernatant were harvested separately. The cells were counted using a blood cell counting chamber, and cell smears were made for Giemsa staining or leucocyte differential count. Lungs were collected for experiments such as histopathological examination and gene expression detection.

### 2.6. Carbon Content in Airway Macrophages (CCAM) Assay

For each mouse, 50 well-stained macrophages with intact envelopes were selected for analysis, and the nuclei were removed before quantification of the areas of carbon particles and cytoplasm to avoid biases derived from different sizes of macrophages and DEP particles overlapping with nuclei. A light microscope (Nikon E200 Japan) equipped with a 100× oil-immersion objective was mounted on a Nanjing Heng Qiao Instrument C630 camera. The obtained images were analyzed using Image J software [23,24].

### 2.7. Masson Staining and Hematoxylin–Eosin (HE) Staining

The left lungs were fixed in 10% neutral formaldehyde for 24 h. The lung tissues were dehydrated, embedded in paraffin, and sliced into 5 μm sections on a rotary microtome. The sections were subjected to HE staining and Masson staining. Histological changes in lung tissues were observed under a light microscope (Nikon, Japan). The obtained images were analyzed using Image J software. Airway circumference was divided by collagen area (the blue area positive for Masson’s staining) around airway, which was calculated for the final “score of collagen deposition” [25].

### 2.8. Immunohistochemistry (IHC)

For immunohistochemical detection of related indicator expression in lungs, 5 μm lung tissues were stained with anti-α-SMA (Proteintech, Wuhan, China, rabbit polyclonal at a 1:1000 dilution) and anti-CXCL17 (ImmunoWay Biotechnology, Suzhou, China, rabbit polyclonal at a 1:100 dilution) antibodies. Next, they were fluorescent-labeled with goat antirabbit IgG (H + L) cross-adsorbed secondary antibody (Zsbio, China).

Areas positively immunostained for CXCL17 or α-SMA proteins in airway were observed under a light microscope (Nikon, Japan). The obtained images were analyzed using Image J software. The intensity of CXCL17 staining was divided into four scores: 0 (no staining), 1 (light brown), 2 (brown), and 3 (dark brown). Positive cells scored based on the percentage of positively stained cells (0–100%). The final CXCL17 score was calculated by multiplying the intensity score by the percentage of positive cells. Airway circumference was divided by airway smooth muscle area (the area positive for α-SMA staining), which was calculated for the final “score of α-SMA staining in airway” [26].

### 2.9. Western Blot Analysis

Proteins of the frozen lung tissues were lysed on ice in RIPA buffer (Beyotime, Shanghai, China) containing proteinase inhibitors (Meilunbio, Dalian, China). The total protein content was evaluated using a Pierce BCA Protein Assay Kit (Beyotime, Shanghai, China). The total proteins were electrophoretic separated and transferred on PVDF membranes (Merck Millipore, Billerica, MA, USA). After blocking for 1 h with bovine serum albumin, the membranes were incubated at 4 °C overnight with primary antibody: Fibronectin (Proteintech, Wuhan, China, 1:1000), Collagen Type I (Proteintech, Wuhan, China, 1:1000), cleaved caspase-3 (Abcam, Cambridge, MA, USA, 1:500), Bax (Cell Signaling, BSN, USA, 1:1000), and GAPDH (Abcam, Cambridge, MA, USA, 1:10,000). After washing with TBST, the membranes were incubated with anti-rabbit IgG secondary antibody (Elibscience, China, 1:2000). The proteins bands were visualized using ECL detection reagents (Shandong Sparkjade Biotechnology Co., Ltd., Jinan, China) and analyzed in chemiluminescence imaging analysis system (Sagecreation, Beijing, China). Densitometry analysis of western blot was quantified via Image J software.

### 2.10. Quantitative Real-Time Polymerase Chain Reaction

Total RNA of HBE, mice lung, and trachea were extracted by TRIzol (Takara, Japan), and the concentrations of isolated total RNA were determined using a NanoDrop One Spectro-photometer (NanoDrop Technologies, Wilmington, DE, USA). The first strand cDNA synthesis (Reverse Transcription Kit, Takara, Dalian, China) and real-time PCR (SYBR^®^ Green Pro Taq HS, Takara, Dalian, China) were also performed according to the manufacturer’s instructions. The relative quantification of the transcripts was performed after normalization against GAPDH. Primer information is provided in Supplementary Table S2. The relative RNA levels were calculated using 2^−ΔΔCt^ method.

### 2.11. Enzyme-Linked Immunosorbent Assay

The levels of CXCL17 (Cusabio Biotech, Wuhan, China) in mice BALF and plasma were detected by double antibody sandwich ELISA according to the manufacturer’s instructions. The minimum detectable dose of mouse CXCL17 was typically less than 9.375 pg/mL.

### 2.12. Cell Lines and Culture

HBE cells (a gift from Prof. Yuxin Zheng, Qingdao University) were preserved in DMEM (Solarbio, Beijing China), and THP-1 cells (Procell, Wuhan, China) were stored in RPMI 1640 medium (Solarbio, Beijing, China) under standard conditions (37 °C, 5% CO_2_/95% air), supplemented with 10% fetal bovine serum (Procell, Wuhan, China), 1% penicillin (Procell, Wuhan, China), and 1% streptomycin (Procell, Wuhan, China). In addition, 10 μg/mL or 20 μg/mL SRM 2975 (the commercial DEP matter, the National Institute of Standards and Technology, Gaithersburg, MD, USA) were added into the culture medium of HBE cells for 72 h except cell migration assay.

### 2.13. Cell Migration Assay

The cell migration assay was performed using 24-well plates with a membrane pore size of 5 μm (Corning, Amsterdam, NDL). THP-1 cells were seeded in the upper chamber and rested in a PMA-free medium for 24 h after 48 h stimulation with PMA at a concentration of 100 ng/mL. Then, the HBE cells were inoculated into the lower chamber and divided into control, CXCL17, DEP, and DEP + CXCL17 groups according to different stimuli. The HBE cells in the lower chamber of the CXCL17 group were stimulated with 200 ng/mL rhCXCL17 (Biolegend, San Diego, USA) for 48 h. The HBE cells in the lower chamber of the DEP group were stimulated with 20 μg/mL SRM 2975 for 48 h. The HBE cells in the lower chamber of the DEP + CXCL17 group were stimulated with 20 μg/mL SRM 2975 and 200 ng/mL rhCXCL17 for 48 h. Subsequently, the cells in the upper chamber migrating across the membrane were fixed, followed by crystal violet staining for cell counting or Swiss staining for the detection of carbon content in airway macrophages (CCAM). The cells in the lower chamber were collected for assays, including flow cytometry and PCR.

### 2.14. Flow Cytometry

The harvested HBE cells were centrifuged at 300× *g* for 5 min. BD FACS Calibur™ flow cytometer (BD Biosciences, Franklin Lakes, NJ, USA) was running according to the operating instructions. Annexin V-FITC Apoptosis Detection Kit (Elabscience, China) was adopted to assess cell apoptosis as the manufacturer’s protocols. Data were analyzed using FlowJo V10 software.

### 2.15. Statistical Analyses

All statistical analyses were performed with the GraphPad Prism 7 software and presented as means ± SD. The normality of the continuous data was tested using the Shapiro–Wilks test. When data conform to normality and homoscedasticity, the student’s *t*-test was used for comparisons between two groups and one-way analysis of variance. This was followed by a Tukey test, which was applied for multiple group comparisons. For data that did not conform to normality or homoscedasticity, Mann–Whitney or Kruskal–Wallis tests were applied. *p* < 0.05 was considered to be a significant difference.

## 3. Results

### 3.1. Effects of Chronic DEE Exposure on Respiratory Parameters, Airway Inflammation, and Airway Injury and Remodeling in Mice

**Figure 2:**
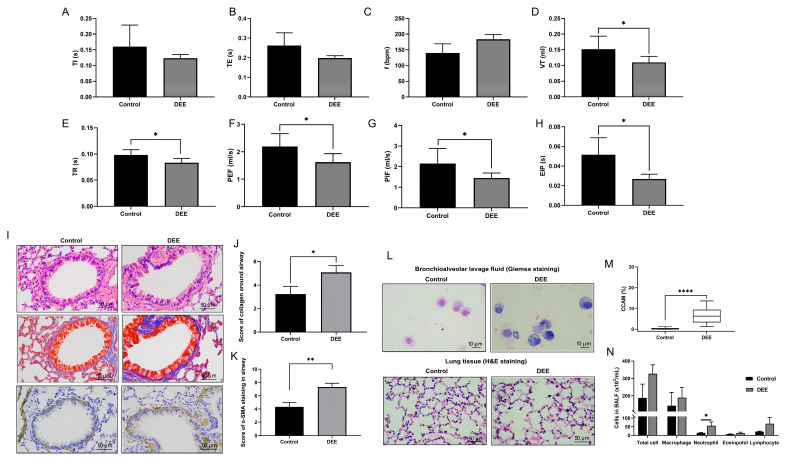
DEE exposure in mice resulted in deterioration of respiratory parameters and airway structure. (**A**–**H**) Respiratory parameters in conscious mice were assessed by whole body plethysmography. TE: expiratory time; TI: inspiratory time; f: Breathing frequency; VT: tidal volume; TR: relaxation time; PEF: peak expiratory flow; PIF: peak inspiratory flow; EIP: end inspiratory pause. (**I**–**K**) HE staining, MASSON staining, and α-SMA immunohistochemical staining were performed on the airway of mice lung tissue; Scale bar = 50 μm. (**L**) Carbon-laden macrophages in BALF (Scale bar = 10 μm) and lung tissue (Scale bar = 50 μm). Arrowheads show carbon laden macrophages. (**M**) Carbon content in airway macrophages. (**N**) Inflammatory cell differential count in BALF. Results are displayed as means ± SD (n = 6 for each group); * *p* < 0.05, ** *p* < 0.01, **** *p* < 0.0001. DEE; diesel exhaust emissions, BALF; bronchoalveolar lavage fluid, DEP; Diesel exhaust particles, CCAM; carbon content in airway macrophages.

The general composition of diesel exhaust atmospheres is presented in Supplementary Table S1. After 3 months of DEE exposure, whole body plethysmography showed worse respiratory parameters in the DEE-exposed mice, as indicated by decreased expiratory time (TE), tidal volume (VT), respiratory flow rates (PIF, PEF, EIP), and airway relaxation time (TR) (Figure 2A–H). As depicted in Figure 2I–K, the disordered arrangement of airway epithelial cells, thickening of peribronchial smooth muscle, and increased collagen were observed in mice after DEE exposure. A large number of DEP particles were phagocytosed by macrophages, and CCAM was significantly higher than the control group (Figure 2L,M). In addition, neutrophils in BALF were markedly elevated in the DEE-exposed mice but not macrophages (Figure 2N). Taken together, the infiltration of airway inflammatory cells, airway injury, and airway remodeling (such as the thickening of peri-bronchial smooth muscle and increased collagen) aggravated mice after DEE exposure; this aggravation was accompanied by worse respiratory parameters.

### 3.2. DEE Exposure Can Downregulate the CXCL17 Expression in Airway Epithelium

**Figure 3:**
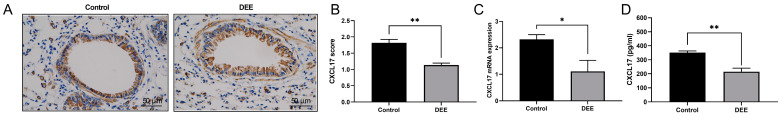
Effects of DEE on CXCL17 expression in mice lung. (**A**,**B**) The protein level of CXCL17 in mouse airway exposed to DEE was detected by IHC, Scale bar = 50 μm. (**C**) CXCL17 mRNA expression in mouse airway exposed to DEE was detected by qRT-PCR. (**D**) CXCL17 protein expression in BALF of mice exposed to DEE was detected by ELISA. Data are displayed as means ± SD (n = 6 for each group) * *p* < 0.05, ** *p* < 0.01. DEE; diesel exhaust emissions, DEP; Diesel exhaust particles.

**Figure 4:**
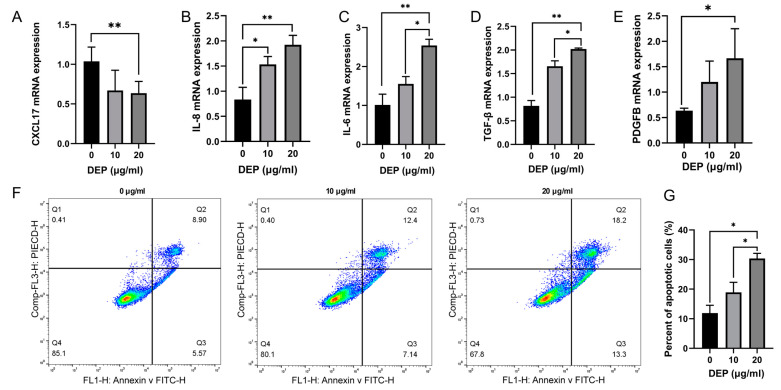
Exposure to DEP increases pro-inflammatory cytokine and apoptosis of HBE cells. (**A**) Expression of CXCL17 mRNA in HBE cells after 72 h of DEP exposure was detected by qRT-PCR. (**B**–**E**) IL-8, IL-6, TGF-β, and PDGFB mRNA expression in DEP-exposed HBE cells was detected by qRT-PCR. (**F**,**G**). Effect of DEP on HBE cell apoptosis was analyzed by flow cytometry. Results are displayed as means ± SD (n = 3 for each group); * *p* < 0.05, ** *p* < 0.01.

CXCL17 score by Image J was significantly decreased in the airway epithelium of the DEE-exposed mice compared with those in the control group (Figure 3A,B). The qRT-PCR and ELISA data exhibited the CXCL17 mRNA expression in the airways and the CXCL17 protein expression in the BALF were also notably reduced in the DEE-exposed mice versus the control mice (Figure 3C,D). Moreover, DEP stimulation could inhibit the expression of CXCL17 mRNA in HBE cells (Figure 4A).

### 3.3. Effects of DEP on Inflammation and Apoptosis of HBE Cells

To evaluate the impacts of DEP exposure on indicators of HBE cell inflammation and remodeling, we determined the mRNA expression of relevant genes by qRT-PCR. Compared with the control group, 10 μg/mL DEP could upregulate the mRNA levels of IL-6, IL-8, and TGF-β, while 20 μg/mL DEP could upregulate PDGFB (platelet-derived growth factor subunit B) mRNA expression (Figure 4B–E). In addition, we evaluated the cell apoptosis in different groups by flow cytometry. The results revealed that 20 μg/mL DEP significantly enhanced the apoptosis of HBE cells, as compared to the control group (Figure 4F,G). Accordingly, 20 μg/mL DEP were used in the following experiments.

### 3.4. CXCL17 Overexpression Promotes Alveolar Macrophage Recruitment and Clearance of DEP in Mice

**Figure 5:**
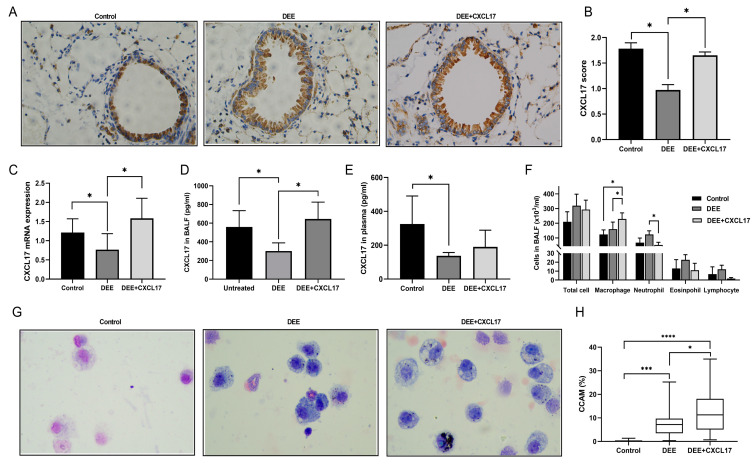
CXCL17.AAV5 upregulated DEE-induced CXCL17 downregulation and enhanced macrophage recruitment and clearance of DEE in mice. (**A**,**B**) The expression of CXCL17 protein in mice airway was detected by IHC. (**C**) CXCL17 mRNA expression in mice airway was detected by qRT-PCR. (**D**) CXCL17 protein expression in BALF was detected by ELISA. (**E**) CXCL17 protein expression in mice plasma was determined by ELISA. (**F**) Inflammatory cell differential count in BALF. (**G**,**H**) Carbon-laden macrophages in BALF and carbon content in airway macrophages. Data are displayed as means ± SD. n = 6. * *p* < 0.05, *** *p* < 0.001, **** *p* < 0.0001. DEE; diesel exhaust emissions, BALF; bronchoalveolar lavage fluid.

Immunofluorescence results showed that AAV5-GFP was successfully infected into the airway epithelium of mice (Supplementary Figure S1). In comparison with the DEE-exposed mice, CXCL17 expression was significantly increased in the airway epithelium and BALF of the AAV5-CXCL17 intervention group (Figure 5A–D) but not in the plasma (Figure 5E). Additionally, significantly increased BALF macrophages (Figure 5F) and CCAM were found in the AAV5-CXCL17 intervention group compared with the DEE exposure group. (Figure 5G,H).

### 3.5. CXCL17 Overexpression Improved Respiratory Parameters and Alleviated Airway Injury and Airway Remodeling in the DEE-Exposed Mice

**Figure 6:**
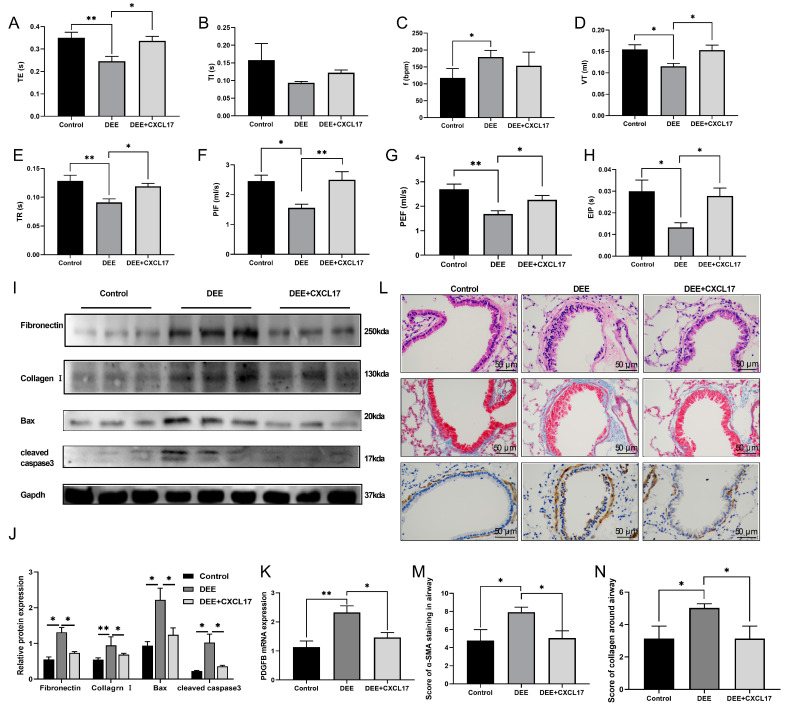
CXCL17.AAV5 improved the respiratory parameters, airway injury, and remodeling in DEE mice. (**A**–**H**) Respiratory parameters in conscious mice were assessed by whole body plethysmography. TE: expiratory time; TI: inspiratory time; f: Breathing frequency, VT: tidal volume; TR: relaxation time; PEF: peak expiratory flow; PIF: peak inspiratory flow; EIP: end inspiratory pause. (**I**,**J**) The protein expression of Fibronectin, Collagen I, Bax, and cleaved caspase 3 in mice lung were measured by Western blot. (**K**) Expression of PDGFB mRNA in mice lung was detected by qRT-PCR. (**L**–**N**) HE staining, Masson staining, and α-SMA immunohistochemical staining were performed on the airway of mice; Scale bar = 50 μm. Data are displayed as means ± SD. n = 6. * *p* < 0.05, ** *p* < 0.01.

WBP results showed that the respiratory parameters (indexes such as TE, VT, PIF, PEF, EIP, TR) were significantly improved in the AAV5-CXCL17 intervention group compared with the DEE group (Figure 6A–H), accompanied by reductions in the levels of several airway remodeling indicators (PDGFB, fibronectin, and Collagen I), as well as airway injury markers (Bax and cleaved caspase3) (Figure 6I–K). Meanwhile, the disordered arrangement of airway epithelial cells and airway remodeling (airway smooth muscle layer thickness and collagen deposition) were also significantly ameliorated in the AAV5-CXCL17 intervention group. (Figure 6L–N).

### 3.6. CXCL17-Mediated Macrophage Can Alleviate DEP-Induced HBE Cell Injury

**Figure 7:**
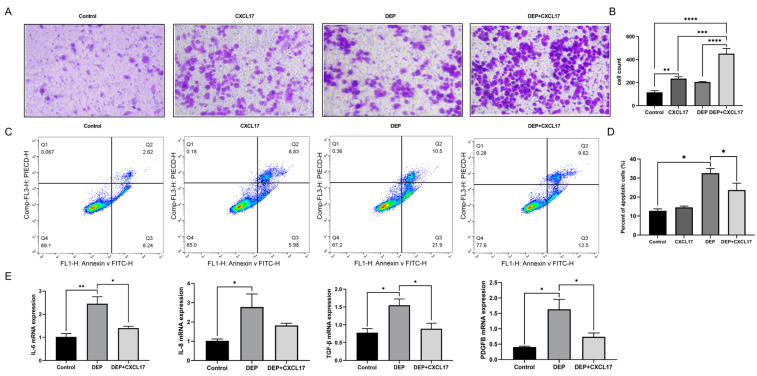
rhCXCL17 can recruit macrophages and alleviate the damage of HBE cells caused by DEP. (**A**,**B**) Migration of THP-1 cells stimulated by CXCL17 and DEP. (**C**,**D**) Apoptosis of HBE cells was analyzed by flow cytometry. (**E**) The mRNA expression of IL-6, IL-8, TGF-β, and PDGFB in HBE cells was detected by qRT-PCR. Data are displayed as means ± SD. (n = 3). * *p* < 0.05, ** *p* < 0.01, *** *p* < 0.001, **** *p* < 0.0001. DEP; Diesel exhaust particles.

The results of the transwell assay showed that THP-1 cell migration remarkably increased in the DEP + CXCL17 group when compared with the DEP group (Figure 7A,B). The flow cytometry results showed that rhCXCL17 intervention could inhibit DEP-induced apoptosis of HBE cells (Figure 7C,D). The qRT-PCR results showed that the levels of an inflammatory factor (IL-6) and remodeling indexes (TGF-β and PDGFB) in HBE cells were significantly reduced in the DEP + CXCL17 group as compared to the DEP group (Figure 7E–H).

## 4. Discussion

As an important public health problem, DEE exposure-related lung injury has attracted increasing attention, yet the underlying mechanisms remain unknown. This was the first study to investigate the role and possible mechanism of macrophage chemokine CXCL17 in DEE-caused lung injury. Our study found that DEE led to aggravated airway injury, remodeling, and respiratory parameters in mice, accompanied by the downregulation of CXCL17 expression in the airway epithelium. Upregulation of CXCL17 in vitro and in vivo could recruit macrophages for the airways and attenuate airway epithelial injury and airway remodeling, which might be associated with corresponding changes in inflammation and remodeling indicators (IL-6, IL-8, TGF-β, PDGFB, etc.).

DEE has been an important environmental pollutant, and its inhalation can initiate and aggravate several respiratory diseases. Several epidemiological studies have reported a higher incidence of asthma among children in high DEE exposure areas, as well as a higher frequency of hospitalization for asthma and COPD exacerbation among long-term DEE-exposed populations [27,28,29]. Our prior study revealed that small airway walls were significantly thickened in the workers under chronic exposure to diesel exhaust [30]. In this study, we found that mice exposed to DEE developed airway epithelial damage, airway smooth muscle hyperplasia, and excessive peribronchial collagen deposition, which was accompanied by worse respiratory parameters (reduced respiratory flow rate, tidal volume, airway relaxation time, etc.). While in vitro, DEP significantly increased the apoptosis of HBE cells. Thus, we could conclude that DEE exacerbated airway injury and remodeling, but the underlying mechanism remained unclear.

The present study demonstrated that the deposition of DEP in the lungs could induce inflammation and oxidative stress, ultimately leading to damage to lung tissues [6,7]. As the first line of innate immune defense, macrophages in the lung can phagocytose and eliminate environmental particulate matter. A prior study reported that multiple inflammatory cells, such as macrophages and neutrophils, were increased in the mouse lungs following acute DEE exposure, while no significant changes were noted in inflammatory cells in the mouse lungs after chronic exposure [31]. Similarly, we found that the macrophages in the BALF tended to be elevated without statistical significance in the mice after 3-month DEE exposure. More importantly, it has been shown that the phagocytic rate and phagocytic index of macrophages are significantly reduced after exposure to certain particulate matter, suggesting inhibited phagocytic activity [8,9,10,11,32]. However, DEP can form aggregates of different sizes and subsequently impair the phagocytic ability of alveolar macrophages, resulting in deleterious health effects [33]. Although macrophages are essential for the clearance of environmental particulate matter, there are currently no studies on the amelioration of DEE-induced lung injury by enhancing the number and function of macrophages in the lung.

As a new member of the CXC family, CXCL17 is abundantly expressed in the mucosa of the respiratory and gastrointestinal tracts, and it functions in chemotaxis, immune homeostasis, and tumor immune response. However, its receptors are not yet identified [16,34]. CXCL17 can promote the recruitment of specific immune cells (monocytes/macrophages, dendritic cells) to the mucosal barrier, playing a significant role in the formation of the mucosal immune system. Burkhardt’s study suggested that alveolar macrophages were significantly deficient in CXCL17^−/−^ mice, but the recruitment of macrophages to other tissues was not affected [20]. Thus, CXCL17 might play an important role in the recruitment of macrophages in the lungs.

To clarify the role of CXCL17 in DEE-induced lung injury, we performed a series of experiments. Firstly, based on molecular biological experiments, we found that CXCL17 was mainly expressed in the airway epithelium of mice. Meanwhile, CXCL17 expression was markedly reduced in both lung tissues and BALF of DEE-exposed mice. In vitro, DEP stimulation downregulated CXCL17 mRNA expression in HBE cells. Up to now, we demonstrated for the first time that DEE reduced CXCL17 expression in the airway epithelium. Subsequently, we constructed a CXCL17 overexpression virus vector (AAV5-CXCL17) and introduced it into the airways to observe its effect on lung injury in the DEE-exposed mice. We found that airway injury/remodeling (airway epithelial damage, airway smooth muscle hyperplasia, peribronchial collagen deposition, and related molecular markers, etc.) and disturbed respiratory parameters (VT, PEF, PIF, TR, etc.) in DEE mice were significantly improved by CXCL17 overexpression. Additionally, mice with CXCL17 overexpression in the airways had significantly increased macrophages in the BALF as well as more CCAM, indicating stronger phagocytic activity of macrophages. It was suggested that CXCL17 potentially protected against DEE-related lung injury by recruiting alveolar macrophages and enhancing their phagocytic function. To further validate the above conclusion, we constructed an in vitro macrophage–airway epithelial cell (THP-1/HBE) co-culture system. Our study found that rhCXCL17 stimulation boosted the migration of THP-1 cells and the phagocytosis of DEP (induced an increase of CCAM), and it alleviated the apoptosis of HBE cells caused by DEP.

Abnormal expression of inflammatory cytokines and growth factors in the lung is closely related to the pathogenesis of multiple respiratory diseases. For instance, the levels of inflammatory cytokines such as IL-6 and IL-8 are remarkably increased in the lung tissues and plasma of COPD patients, which are significantly correlated with disease severity and exacerbation frequency [35,36,37,38,39,40]. Furthermore, several studies have revealed that inhibiting the expression of the above can alleviate lung injury in COPD mice [41,42]. Existing studies have documented that IL-6 and IL-8 are increased in the sputum and plasma of DEE-exposed subjects and their lung function declined [43,44]. Furthermore, we found that the mRNA levels of IL-6, IL-8, and other inflammatory genes were markedly increased in the lung tissues of the DEE-exposed mice, which significantly decreased after CXCL17 overexpression in the airways. Similarly, our in vitro data showed that rh-CXCL17 recruited macrophages and inhibited DEP-induced mRNA expression of inflammatory genes such as IL-6 and IL-8 in HBE cells. Therefore, it was reasonable to presume that CXCL17-induced alleviation of DEE-associated airway injury might be related to the recruitment of macrophages and activation of their phagocytic function, which in turn reduced the expression of inflammatory factors such as IL-6 and IL-8.

TGF-β and PDGFB are common growth factors involved in the development of multiple pulmonary diseases such as pulmonary fibrosis, pulmonary hypertension, and COPD [45,46]. Studies have shown that the expression of TGF-β and PDGFB is markedly increased in patients with the above diseases, and inhibiting the expression of the aforementioned genes using pharmacological or genetic interventions can suppress the remodeling of airways or blood vessels, etc. [47,48,49,50]. Our study found that the mRNA levels of TGF-β and PDGFB increased notably in the lungs of DEE mice, accompanied by increased expression of remodeling markers including fibronectin and Collagen I. However, the above effects of DEE were inhibited by CXCL17 overexpression. More importantly, rh-CXCL17 could recruit macrophages in vitro and inhibit DEP-induced upregulation of TGF-β and PDGFB in HBE cells. Therefore, we speculated that the inhibitory impact of CXCL17 on DEE-associated airway remodeling might be related to the downregulation of growth factors (such as TGF-β and PDGFB) caused by increased “macrophage recruitment” and “activation of their phagocytic function”.

There are several limitations to this study. Firstly, owing to technical limitations, this study only examined the external exposure level of DEE for mice, but not the internal exposure levels of DEE. Secondly, this study focused on the therapeutic significance of CXCL17 overexpression in the airways for DEE-induced lung injury, while its preventive effect and co-acting receptors need to be further explored.

## 5. Conclusions

CXCL17 overexpression could attenuate DEE-induced lung injury by recruiting lung macrophages and activating their phagocytic function. In addition, CXCL17 may serve as a new therapeutic target gene for DEE-related pulmonary diseases.

## Data Availability

The data used to support the findings of this study are available from the corresponding author upon request.

## Supplementary Materials

The following supporting information can be downloaded at: https://www.mdpi.com/article/10.3390/toxics11080646/s1, Table S1: Concentration of different pollutants from diesel exhaust emissions; Table S2: primer sequences; Figure S1: Immunofluorescence staining on the airway of mice lung tissue.
