# Supplementary material for: CXCL17 Attenuates Diesel Exhaust Emissions Exposure-Induced Lung Damage by Regulating Macrophage Function
Source: Toxics. 2023 Jul 26;11(8):646. doi: 10.3390/toxics11080646 (PMC10459829; doi:10.3390/toxics11080646)
Supplement: Supplementary file 1 [file toxics-11-00646-s001.zip › toxics-2463116-supplementary.pdf]

Supplementary Material

1 Supplementary Figures and Tables

1.1 Supplementary Tables

supplementary Table S1. Concentration of different pollutants from diesel exhaust emissions

Parameter	Value
PM (mg/m^3)	3.156
NO_X (ppm)	18.54
CO (ppm)	10.37

PM; particulate matter, NOx; oxides of nitrogen, CO; carbon monoxide. Data are shown as mean.

supplementary Table S2. primer sequences

**Gene**	**Forward**	**Reverse**
CXCL17-mus	GGAAGGTGGCCAAGAATGTGA	GATCACAGGGACACTGCTTCC
IL6-mus	CTTCTTGGGACTGATGCTGGTGAC	TCTGTTGGGAGTGGTATCCTCTGTG
IL-8-mus	GGCAAGAACACTGTGTCCAAAGA	GTGGATGACTGTCCATGCAGAA
TGF-β-mus	TTCGATTCAGCGCTCACTGCT	CGGTTCATGTCATGGATGGTGC
PDGFB-mus	CATCCGCTCCTTTGATGATCTT	GTGCTCGGGTCATGTTCAAGT
GAP-mus	CATCACTGCCACCCAGAAGACTG	ATGCCAGTGAGCTTCCCGTTCAG
CXCL17-homo	CGGCCAAGAATGTGAGTGCAA	TGTGGTGCCTTTGGTGTCTTG
IL-6-homo	ACTCACCTCTTCAGAACGAATTG	CCATCTTTGGAAGGTTCAGGTTG
IL-8-homo	ACTGAGAGTGATTGAGAGTGGAC	AACCCTCTGCACCCAGTTTTC
TGF-β-homo	TACAGCAACAATTCCTGGCGATACC	CTCAACCACTGCCGCACAACTC
PDGFB-homo	TCCCGAGGAGCTTTATGAGA	ACTGCACGTTGCGGTTGT

GAPDH-homo	CAACGTGTCAGTGGTGGACCTG	GTGTCGCTGTTGAAGTCAGAGGAG

1.2 Supplementary Figures

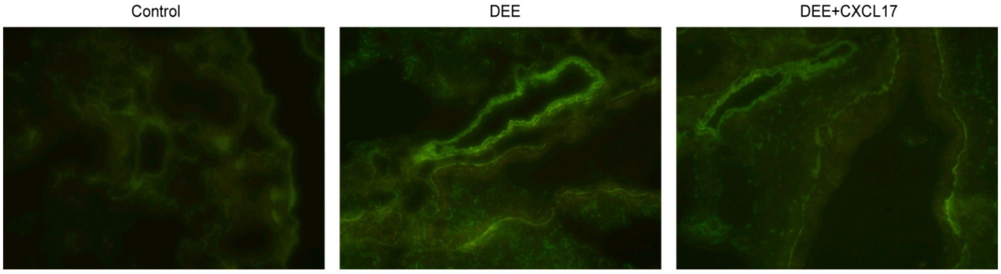

Supplementary Figure S1. Immunofluorescence staining on the airway of mice lung tissue
